# Supplementary material for: Modifications to student quarantine policies in K–12 schools implementing multiple COVID-19 prevention strategies restores in-person education without increasing SARS-CoV-2 transmission risk, January-March 2021
Source: PLoS One. 2022 Oct 20;17(10):e0266292. doi: 10.1371/journal.pone.0266292 (PMC9584452; doi:10.1371/journal.pone.0266292)
Supplement: S1 Table — Abbreviations: K–12 = kindergarten through grade 12; NR = not reported. Note: Data are from surveys completed by school and district officials unless otherwise noted. * Data from: https://dese.mo.gov/school-data. † Includes participating schools only. ‡ Race and ethnicity categories at the school level differed from those collected from individuals as part of the investigation. (DOCX) [file pone.0266292.s001.docx]

**S1 Table**. Characteristics of 103 public K–12 schools participating in SARS-CoV-2 transmission investigation, Greene and St. Louis Counties, Missouri, January 25–March 21, 2021.

| **District characteristic** | **A** | **B** | **C** | **D** | **E** | **F** |
| --- | --- | --- | --- | --- | --- | --- |
| County | Greene | Greene | Greene | St. Louis | St. Louis | St. Louis |
| District size* (sq. mi.) | 137.9 | 100.3 | 122.8 | 157.2 | 25.2 | 6.2 |
| Primary settings | Urban, Suburban | Suburban, Rural | Suburban, Rural | Urban, Suburban | Urban, Suburban | Urban, Suburban |
| Number of schools^†^ | 45 | 7 | 5 | 30 | 9 | 7 |
| Total number of students | 23,029 | 4,828 | 2,240 | 20,716 | 5,791 | 2,450 |
| % students 100% virtual | 28 | 5 | 3 | 18 | 43 | 55 |
| % student population by race and ethnicity^‡^ |  |  |  |  |  |  |
| Asian, non-Hispanic/Latino | 3 | 1 | 1 | 10 | 4 | 1 |
| Black or African American, non-Hispanic/Latino | 8 | 1 | <1 | 8 | 34 | 80 |
| Hispanic/Latino | 8 | 5 | 2 | 4 | 11 | 4 |
| White, non-Hispanic/Latino | 74 | 79 | 89 | 75 | 42 | 11 |
| Other | 6 | 5 | 3 | 4 | 9 | 4 |
| Unknown | 0 | 8 | 4 | 0 | 0 | 0 |
| % students eligible for free or reduced-price school meals | 48 | 41 | 33 | 12 | 35 | 100 |
| Total number of staff | 1,702 | 528 | 500 | 2,871 | 640 | 326 |
| % staff 100% virtual | 0 | NR | 40 | 2 | 11 | NR |

Abbreviations: K–12 = kindergarten through grade 12; NR = not reported

Note: Data are from surveys completed by school and district officials unless otherwise noted.

* Data from: <https://dese.mo.gov/school-data>

† Includes participating schools only.

‡ Race and ethnicity categories at the school level differed from those collected from individuals as part of the investigation.
