# Supplementary material for: Modifications to student quarantine policies in K–12 schools implementing multiple COVID-19 prevention strategies restores in-person education without increasing SARS-CoV-2 transmission risk, January-March 2021
Source: PLoS One. 2022 Oct 20;17(10):e0266292. doi: 10.1371/journal.pone.0266292 (PMC9584452; doi:10.1371/journal.pone.0266292)
Supplement: S2 Table — Abbreviations: COVID-19 = coronavirus disease 2019; K–12 = kindergarten through grade 12; RR = relative risk; CI = confidence interval. Note: All regression models accounted for cluster-correlated observations at the school level using generalized estimating equations (GEE) with an independent correlation matrix. * Regression models were adjusted for school role (student versus staff). † Surveyed ventilation strategies included opening doors when possible, opening windows when possible, using fans to circulate air, and updating heating, ventilation, and air conditioning (HVAC) systems specifically to prevent COVID-19. (DOCX) [file pone.0266292.s002.docx]

**S2 Table**. Unadjusted and adjusted relative risks of school-based SARS-CoV-2 infection among school close contacts by schoolwide COVID-19 policy in K–12 schools, Greene and St. Louis Counties, Missouri, January 25–March 21, 2021.

| **Schoolwide policy** | **Crude RR (95% CI)** | **Adjusted* RR (95% CI)** |
| --- | --- | --- |
| Quarantine |  |  |
| Modified quarantine | 0.71 (0.22–2.32) | 0.76 (0.24–2.38) |
| Standard quarantine | Referent | Referent |
|  |  |  |
| Mode of instruction |  |  |
| Virtual instruction available | 0.94 (0.16–5.61) | 0.89 (0.17–4.77) |
| In-person instruction only | Referent | Referent |
|  |  |  |
| Face mask mandate |  |  |
| Enforced with disciplinary action | 0.22 (0.06–0.91) | 0.29 (0.08–1.06) |
| Not enforced with disciplinary action | Referent | Referent |
|  |  |  |
| Ventilation strategy^†^ |  |  |
| Applied 3-4 strategies | 1.45 (0.45–4.68) | 1.37 (0.44–4.32) |
| Applied 0-2 strategies | Referent | Referent |
|  |  |  |
| Physical barriers |  |  |
| Used in some/all classrooms | 0.32 (0.08–1.36) | 0.35 (0.10–1.30) |
| Not used in classrooms | Referent | Referent |
|  |  |  |
| Cohorting/podding of students |  |  |
| Done | 1.12 (0.35–3.57) | 0.97 (0.31–3.05) |
| Not done | Referent | Referent |

Abbreviations: COVID-19 = coronavirus disease 2019; K–12 = kindergarten through grade 12; RR = relative risk; CI = confidence interval

Note: All regression models accounted for cluster-correlated observations at the school level using generalized estimating equations (GEE) with an independent correlation matrix.

* Regression models were adjusted for school role (student versus staff).

† Surveyed ventilation strategies included opening doors when possible, opening windows when possible, using fans to circulate air, and updating heating, ventilation, and air conditioning (HVAC) systems specifically to prevent COVID-19.
