# Supplementary figures and images for: Modifications to student quarantine policies in K–12 schools implementing multiple COVID-19 prevention strategies restores in-person education without increasing SARS-CoV-2 transmission risk, January-March 2021
Source: PLoS One. 2022 Oct 20;17(10):e0266292. doi: 10.1371/journal.pone.0266292 (PMC9584452; doi:10.1371/journal.pone.0266292)

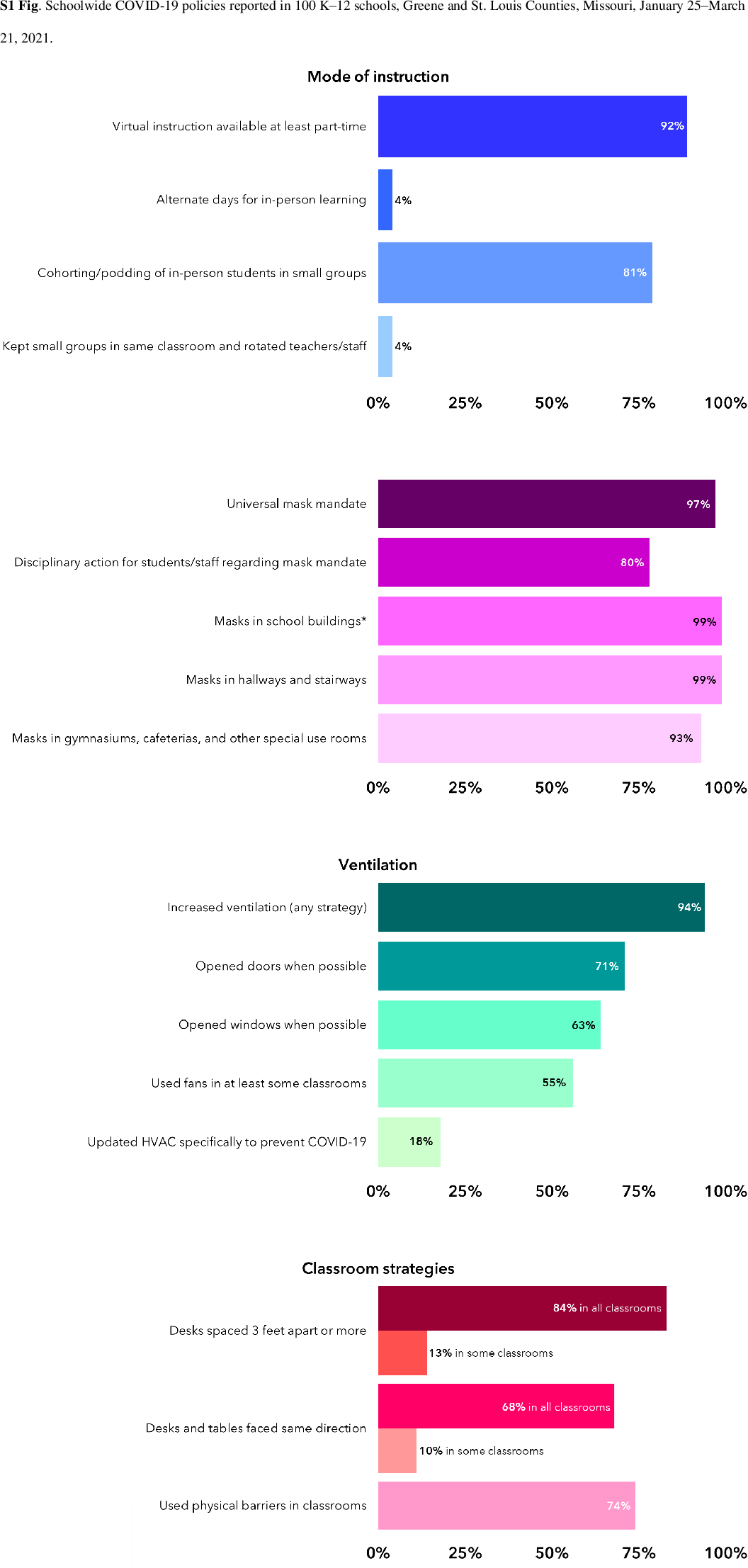

Supplement: S1 Fig — (A-D) Schoolwide COVID-19 policies reported in 100 K–12 schools, Greene and St. Louis Counties, Missouri, January 25–March 21, 2021. Abbreviations: COVID-19 = coronavirus disease 2019; HVAC = heating, ventilation, and air conditioning; K–12 = kindergarten through grade 12. * School buildings also include the areas listed in the subsequent two categories: hallways, stairways, gymnasiums, cafeterias, and other special use rooms. (TIF) [file pone.0266292.s003.tif]
